# Efficacy and safety of chimeric antigen receptor T cell therapy in relapsed/refractory diffuse large B-cell lymphoma with different HBV status: a retrospective study from a single center

**DOI:** 10.3389/fimmu.2023.1200748

**Published:** 2023-05-24

**Authors:** Danqing Kong, Nana Ping, Xin Gao, Rui Zou, Peng Wang, Depei Wu, Zhengming Jin, Changju Qu

**Affiliations:** ^1^ National Clinical Research Center for Hematologic Diseases, Jiangsu Institute of Hematology, The First Affiliated Hospital of Soochow University, Suzhou, China; ^2^ Institute of Blood and Marrow Transplantation, Collaborative Innovation Center of Hematology, Suzhou University, Suzhou, China; ^3^ Department of Gastroenterology, The First Affiliated Hospital of Soochow University, Suzhou, China

**Keywords:** diffuse large B cell lymphoma, hepatitis B virus, chimeric antigen receptor T cell therapy, HBV reactivation, cirrhosis

## Abstract

**Background:**

Chimeric antigen receptor T cell (CAR-T) therapy is an effective salvage treatment in relapsed or refractory(r/r) diffuse large B-cell lymphoma (DLBCL), but the impact of hepatitis B virus (HBV) infection has not been studied.

**Methods and results:**

Here, 51 patients with r/r DLBCL receiving CAR-T therapy were enrolled and analyzed at the First Affiliated Hospital of Soochow University. The overall response rate and the complete remission rate (CR) of CAR-T therapy were 74.5% and 39.2%, respectively. With a median follow-up of 21.1 months after CAR-T, the probabilities of overall survival (OS) and progression-free survival (PFS) at 36 months were 43.4% and 28.7%, respectively. These patients were divided into three cohorts including chronic HBV infection group (n=6), resolved HBV infection group (n=25) and non-HBV infection group (n=20). Bone marrow involvement was significantly higher in the HBV infection group(*P*<0.001), other basic characteristics before CAR-T therapy were comparable. Subgroup analysis showed that HBV infection status did not affect the efficacy of CAR-T therapy in CR rate, OS or PFS, and there was no significant difference in CAR-T related toxicities between three cohorts. Only one cirrhosis patient with chronic HBV infection experienced HBV reactivation.

**Conclusions:**

CAR-T therapy was effective and can be used safely in r/r DLBCL with HBV infection under proper monitoring and antiviral prophylaxis.

## Introduction

Diffuse large B-cell lymphoma (DLBCL) is one of the most common B-cell malignancies. Hepatitis B virus (HBV) infection in patients with DLBCL is not rare and has a higher prevalence than the general population. DLBCL patients with HBV infection showed distinct clinical and molecular characteristics (1). HBV reactivation during immunochemotherapy can result in multiple clinical manifestations, ranging from asymptomatic detectable HBV-deoxyribonucleic acid (DNA) or hepatitis to fatal hepatic failure. In the rituximab era, hepatitis B surface antigen(HBsAg) positivity was an independent unfavorable prognostic factor (2). Increased awareness and antiviral prophylaxis had significantly reduced the rate of HBV reactivation (3, 4).

Chimeric antigen receptor T cell (CAR-T) therapy had been shown to be safe and effective in relapsed/refractory(r/r) B cell malignancies, including DLBCL and about 40% even achieve sustained response (5, 6). However, CAR-T therapy may induce a higher HBV reactivation rate in the presence of B-cell aplasia. Whether CAR-T therapy is applicable in r/r DLBCL with HBV infection is still unclear. Few studies have reported the efficacy and safety of CAR-T therapy in patients with HBV infection, but often involved multiple types of B malignancy diseases or small-scale reports without contrast (7–10). Herein, our respective cohort study focused on DLBCL patients to assess the safety and efficacy of CAR-T therapy with different HBV infection status.

## Patients and methods

### Patients

We conducted a phase II clinical trial that tested the efficacy and toxicities of CAR-T therapy in patients with r/r non-Hodgkin’s lymphoma (NCT03196830) at the First Affiliated Hospital of Soochow University. Among the patients enrolled, a total number of 51 patients with r/r DLBCL who received CAR-T therapy from Jan,2018 to Oct,2021 were drawn and analyzed. The diagnosis was made on the basis of the World Health Organization classification. The follow-up visit was conducted until death or the last visit time June 1, 2022.

### Monitoring of HBV infection

Markers related to HBV infection including HBV serology [HBsAg, hepatitis B surface antibody (HBsAb), hepatitis B e antigen (HBeAg), hepatitis B e antibody (HBeAb), hepatitis B core antibody (HBcAb)], HBV-DNA and liver function were closely monitored during lines of therapy in all patients and during follow-up time in chronic and resolved HBV infectious patients. The lower limit quantification of HBV-DNA was 60 international units (IU)/milliliter(mL) based on our laboratory standards. Chronic HBV infection was defined by the detection of HBsAg for more than 6 months. Resolved HBV infection was defined as HBsAg negative, HBcAb positive, HBsAb negative or positive, with undetectable HBV-DNA. HBV reactivation in chronic or resolved HBV infection patients was defined as one of the followings:(1) ≥2 log (100 fold) increase in HBV-DNA compared to the baseline level of HBV-DNA. (2) newly detectable HBV-DNA. (3) reappearance of HBsAg. The hepatitis flare was reasonably defined as alanine aminotransferase (ALT)/aspartate aminotransferase (AST) increase to ≥3 upper limit of normal.

### CAR-T therapy procedures

Patients received CAR-T therapy targeting CD19(n=8), CD19 combined with CD20(n=7), CD19 combined with CD22(n=9) or tandem CD19/CD22 (n=27), CAR-T cells were produced and quality controlled by the Unicar-Therapy Bio-medicine Technology Co. (Shanghai, China) as previously described (11). The detailed procedure was described in the supplementary data S1. Prior to CAR-T infusion, patients received FC (fludarabine 30mg/m2/d, day -5, -4, -3; cyclophosphamide 300mg/m2/d, day -5, -4, -3) or decitabine (DAC a total dose of 100mg/m2 was administrated equally intravenously for 3 consecutive days) bridging FC conditioning regimen for lymphodepletion, and then scheduled infusion of CAR-T cells by dose escalation in 1 to 4 days (decided by physical status of patients and tumor burden), detailed in supplementary data S2. The evaluation included complete blood count (CBC), coagulation routine, organ function, inflammatory markers such as ferritin, cytokines involving interleukin(IL)-2, IL-4, IL-6, IL-10, tumor necrosis factor(TNF), interferon(IFN)-γ,IL-17. Expansion and persistence of CAR-T cells in peripheral blood were monitored after infusion of CAR-T cells. Cytokine release syndrome (CRS) and immune effector cell-associated neurotoxicity syndrome (ICANS) were evaluated based on the widely accepted CRS scoring system (12). The application of corticosteroids or IL-6 receptor antibody tocilizumab and other supportive measures depended on the grade of CRS and ICANS.

### Outcomes assessments and measurement

The response criteria were defined in accordance with the National Comprehensive Cancer Network (NCCN) B-cell lymphomas guidelines, version 4.2022. Primary refractory disease was defined as refractory to first-line immunochemotherapy. Overall survival (OS) was calculated from the first CAR-T infusion to the date of death, or the last follow-up, while progression-free survival (PFS) was calculated from the day of remission after CAR-T therapy to progression, relapse, death, or the last follow-up. Adverse events were evaluated according to the National Cancer Institute (NCI) Common Terminology Criteria for Adverse Events (CTCAE, Version 4.0).

### Statistical analysis

Patient characteristics were summarized using the median and range for continuous variables, and frequency and percentage for categorical variables. Variables were compared between cohorts using Fisher’s exact or chi-square test for categorical variables and Wilcoxon rank-sum test for continuous variables. OS and PFS were estimated by the Kaplan-Meier method and compared using the log-rank test. Statistical analysis was performed using GraphPad Prism 8. The *P* value <0.05 was considered statistically significant.

## Results

### Patient characteristics and antiviral therapy

**Figure 1:**
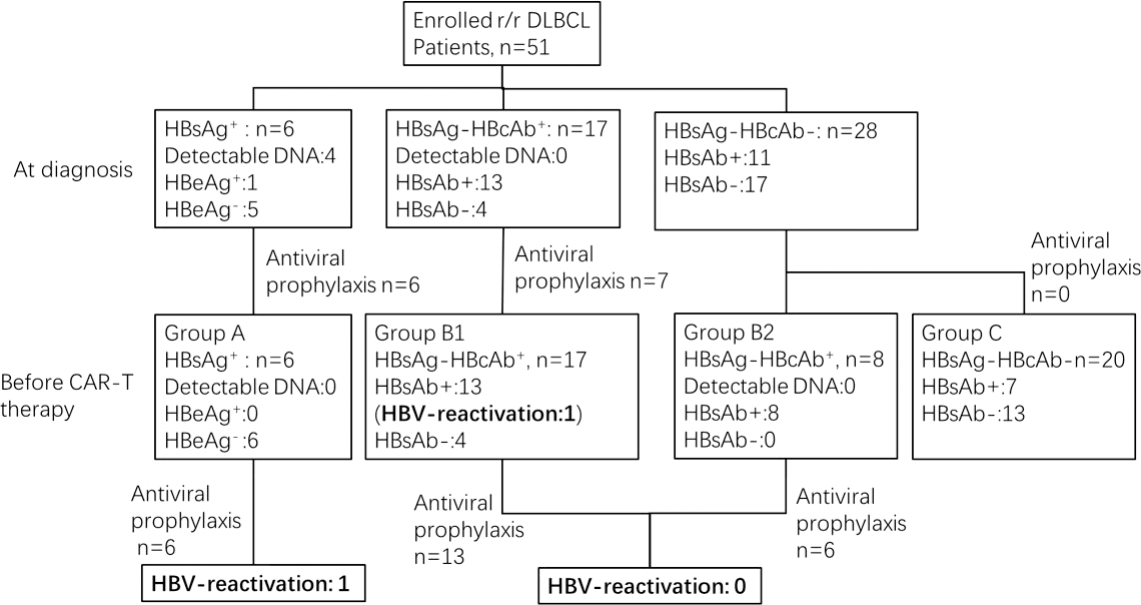
HBV infection status of enrolled patients and antiviral prophylaxis.

51 patients with r/r DLBCL were enrolled in our study. HBV serology at diagnosis of DLBCL and at the time of CAR-T infusion is shown in **Figure 1**. These 51 patients were divided into 3 cohorts based on the status of HBV infection before CAR-T cell infusion as follows: chronic HBV infection group (Group A, n=6), resolved HBV infection group (Group B, n=25) and non-HBV infection group (Group C, n=20).

Among the 6 patients in Group A, four had detectable HBV-DNA at diagnosis. The median time from first detection of HBV DNA/HBsAg seropositivity to infusion of CAR T-cells was 19.9 months (range 4.9 to 56.1 months). After antiviral treatment with entecavir 0.5mg/d or combine adefovir 10mg/d with entecavir 0.5mg/d, HBV-DNA was undetectable before CAR-T infusion. The median time between undetectable for HBV-DNA and CAR-T infusion was 9.2 months (range 4.5 to 13.1 months). Antiviral treatment was continued during and after CAR-T therapy until the last follow-up. Among 25 patients in Group B, 17 patients (Group B1) were diagnosed at baseline, but the other 8 patients (Group B2) were all negative for HBsAg and HBcAb at baseline, and were diagnosed seropositive for HBcAb during chemotherapy. The median time from first detection of HBcAb seropositivity to infusion of CAR T-cells was 7.1 months (range 0.2 to 38.8 months). 19 of them received antiviral prophylaxis with entecavir. HBV serology was monitored during and after CAR-T therapy in all patients.

The basic characteristics of the three groups were comparable, with respect to patient age (including older patients ≥65 years), gender, Ann Arbor III-IV stage, lactate dehydrogenase (LDH), Extra nodal or central nervous system(CNS) involvement at diagnosis, number of lines prior to CAR-T, previous autologous stem cell transplantation(ASCT), use of CD20 antibody and primary refractory disease. However, bone marrow involvement was significantly higher in patients with chronic HBV infection(*P*<0.001). Details are shown in **Table 1**.

**Table 1:** Characteristics of 51 patients with r/r DLBCL prior to CAR-T therapy.

	Group A (n=6)	Group B (n=25)	Group C (n=20)	*P* value
Median age, years (range)	55.3 (44-66)	57.0 (34-72)	52.9 (33-72)	0.41
Age≥65years (n,%)	1 (16.7%)	6 (24%)	3 (15%)	0.74
Male gender (n,%)	5 (83.3%)	13 (52%)	11 (55%)	0.37
Stage III-IV at diagnosis (n,%)	6 (100%)	20 (80%)	17 (85%)	0.48
Median LDH at diagnosis, U/L (range)	240.1 (167-361)	299.9 (98-803)	302.3 (129-917)	0.74
Bone marrow involvement at diagnosis (n,%)	5 (83.3%)	1 (4%)	3 (15%)	<0.001
Extra nodal or CNS involvement at diagnosis (n,%)	2 (33.3%)	12 (48%)	7 (35%)	0.62
Median number of lines prior to CAR-T (range)	3 (2-5)	3.3 (2-7)	3.1 (2-6)	0.59
Transformed DLBCL	1 (16.7%)	3 (12%)	1 (5%)	0.61
Previous ASCT	2 (33.3%)	5 (20%)	3 (14.3%)	0.61
Previous use of CD20 antibody (n,%)	5 (83.3%)	25 (100%)	18 (90%)	0.18
Primary refractory after first line of therapy (n,%)	3 (50%)	9 (36%)	4 (20%)	0.30

LDH for lactate dehydrogenase, CNS for central nervous system, CAR-T for chimeric antigen receptor T cell therapy, DLBCL for diffuse large B-cell lymphoma, ASCT for autologous stem cell transplantation

### Clinical efficacy of CAR-T therapy and survival

**Figure 2:**
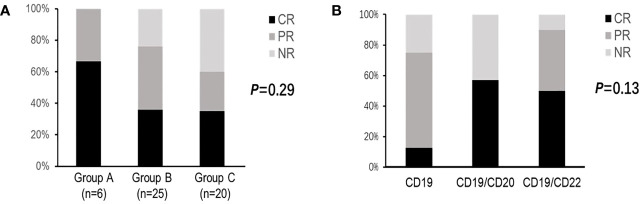
Clinical responses of CAR-T therapy in DLBCL patients. **(A)** Clinical responses in patients with different HBV status. **(B)** Clinical responses in patients with different CAR-T targets. CR for complete response; PR for partial response; NR for no response; CAR-T for chimeric antigen receptor T cell therapy; DLBCL for diffuse large B-cell lymphoma; HBV for hepatitis B virus.

Among the 51 patients, the overall response rate and complete remission(CR) rate of CAR-T therapy were 74.5% and 39.2%, respectively. No significant difference were observed between three groups with different HBV status (*P*=0.29, described in **Figure 2A**). We also conducted subgroup analysis based on different CAR-T targets, patients were divided into CD19 group(n=8,five in Group B and three in Group C),CD19/CD20 group(n= 7, three in Group B and four in Group C)and CD19/CD22 group(n=36, six in Group A, seventeen in Group B and thirteen in Group C). It seemed dual targets CD19/CD22 achieved better response rates, but there were no statistic difference observed (*P*=0.13, described in **Figure 2B**).

**Figure 3:**
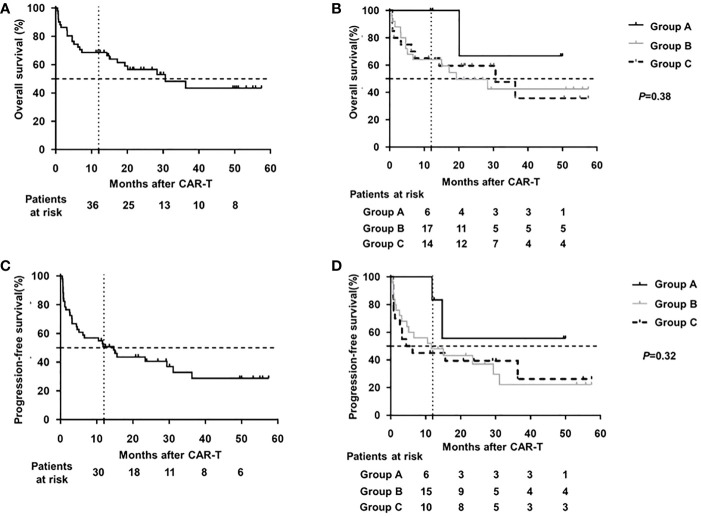
Survival analysis of DLBCL patients after CAR-T therapy **(A)** OS of total cohort. **(B)** OS of different HBV subgroup. **(C)** PFS of total cohort. **(D)** PFS of different HBV subgroup OS for overall survival; PFS for progression-free survival; CAR-T for chimeric antigen receptor T cell therapy; DLBCL for diffuse large B-cell lymphoma; HBV for hepatitis B virus.

The median OS and PFS were 30.6 and 21.8 months, respectively, with a median follow-up of 21.1 months (range 0.5-57.5 months) post CAR-T therapy (shown in **Figure 3**). The estimated probabilities of OS and PFS at 12 months were 68.6% (CI95%: 57.8-83.2%) and 50.8% (CI95%: 38.1-65.1%), respectively. The estimated probabilities of OS and PFS at 36 months were 43.4%(CI95%: 27.3-60.7%) and 28.7% (CI95%: 13.2-42.6%), respectively. No significant differences in OS (*P*=0.38) or PFS (*P*=0.32) were observed among three cohorts.

### Toxicity of CAR-T therapy

The most frequent toxicities associated with CAR-T therapy included CRS, ICANS, organ damage, and hematologic toxicity (**Table 2**). Grade 3-4 CRS was observed in 8 patients and ICANS occurred in 6 patients. Elevations in alanine transaminase (ALT) and total bilirubin (TBIL)were observed in 13 and 3 patients, respectively. 13 patients showed renal dysfunction and 7 patients had heart dysfunction. 46 out of 51 patients showed grade 3-4 hematologic toxicity. In particular, the HBV-infection group did not observe the occurrence of grade 3-4 CRS/ICANS as well as organ damage. Additionally, there were no significant differences in ferritin max/baseline, IL-6 max/baseline, and grade 3-4 hematologic toxicity between three groups.

**Table 2:** Main toxicities of CAR-T therapy.

	Group A (n=6)	Group B (n=25)	Group C (n=20)	*P* value
CRS(Grade 3-4)	0	4	4	0.49
ICANS	0	2 Grade 4:n=2	4 Grade 4: n=2 Grade 2: n=2	0.29
ALT/AST≥2ULN	0	6	7	0.22
TBIL≥1.5ULN	0	2	1	0.74
Renal/heart damage	0	Renal:7 Heart:4	Renal:6 Heart:3	0.31 0.58
Median ferritin max/baseline (range)	2.5 (0.9-3)	4.4 (0.7-12.6)	2.9 (0.5-7.7)	0.07
Median IL-6 max/baseline (range)	9.8 (2.6-53.9)	224.5 (1.28-1226)	17.7 (0.4-198)	0.10
Hematologic toxicity (grade3-4)	6	20	20	0.06

CRS for cytokine release syndrome; ICANS for immune effector cell-associated neurotoxicity syndrome; ALT for alanine transaminase; TBIL for total bilirubin; ULN for upper limit of normal.

In total, 4 patients died from CAR-T therapy, none in Group A, two in Group B (B-17,B-22) and two in Group C(C-33,C-41). Patient B-17 was a 50-year-old female patient who fell into a coma and acute renal failure at day +3 after CAR-T infusion. Unfortunately, she didn’t survive after 6 cycles of continuous renal replacement therapy(CRRT) and died of cardiopulmonary arrest. Patient B-22 was a 72-year-old male patient who developed hypotension and confusion on day +4, and later was associated with recurrent atrial fibrillation, severe pneumonia, and renal failure, and died of multiple organ failure (MOF) 18 days after CAR-T infusion. Patient C-33 was a 40-year-old man who developed hypotension, hypoxemia, and coma on day +5 and recovered with high-dose corticosteroids, tocilizumab, and other supportive management, and he died from septic shock and MOF on day +11. Patient C-41 was a 41-year-old woman, and died of disease progression, recurrent seizures, and MOF on day 14 after CAR-T infusion.

### HBV reactivation and other hepatic complications

In general, HBV reactivation was revealed in 2 patients, one in the chronic HBV infection cohort (patient A-1) after CAR-T therapy and one in the resolved cohort (patient B-11) without prior antiviral prophylaxis before CAR-T therapy. Patient A-1 had B-cell aplasia for 21.2 months, compared to a median duration of 18.5 months (range 11.2-30.7 months) for patients in Group A. Patient B-11 had B-cell aplasia for 17.2 months before HBV reactivation, while the median duration of B-cell aplasia in Group B was 16.8 months (range 6.1-29.5months).

Patient A-1 was a quite interesting case. The 62-year-old man had a history of hepatitis B virus (HBV) infection for more than 30 years, yet paid no attention. When referred to the hospital, liver cirrhosis was manifested with hyperbilirubinemia, hypoalbuminemia, ascites, splenomegaly, and esophageal varices. The serum HBeAg was positive and the HBV-DNA level reached a high up to 3.59×10^7^ IU/mL with mildly elevated ALT. He received entecavir antiviral therapy and eight cycles of immunochemotherapy including rituximab, cyclophosphamide, doxorubicin, vincristine, and prednisone (R-CHOP) and local intensity modulated radiotherapy but still got a rapid disease progression. After anti-CD19 and anti-CD22 CAR-T therapy, he achieved durable DLBCL CR. He remained on antiviral prophylaxis, but no further treatments of DLBCL. But 14 months after CAR-T therapy, his routine check-up by liver enhanced magnetic resonance imaging (MRI) detected newly found nodules with a maximum>2cm in diameter, which led to the diagnosis of hepatic malignant tumor (**Supplementary Figure 1**). Laboratory tests showed that the HBV-DNA level was 94.4 IU/mL, indicating that HBV reactivation occurred, and there was no HBV-related hepatitis flare. After combining antiviral therapy with entecavir and adefovir, together with several sessions of transcatheter arterial chemoembolization and ablation therapy, he survived until the last follow-up, with slowly progressive liver neoplasm and undetectable level of HBV-DNA.

Patient B-11 was a 62-year-old woman with negative HBsAg, positive HBcAb and positive HBsAb, with absence of HBV-DNA, and no antiviral prophylaxis was administered. She achieved CR after 6 cycles of R-CHOP, but soon relapsed. After 2 cycles of salvage combined chemotherapy, including gemcitabine, dexamethasone, and cisplatin (GDP) and 5 cycles of lenalidomide, the disease evaluation was progression of disease (PD). She developed HBV reactivation with a newly detected HBV-DNA titer of 271 IU/mL but no hepatitis flare. She accepted pre-emptive therapy with entecavir and had a quick HBV DNA fall before CAR-T therapy. She did not experience HBV reactivation or liver damage during and after CAR-T therapy up until the last visit.

## Discussion

To our knowledge, this study was the largest cohort study evaluating CAR-T therapy in r/r DLBCL patients with different HBV status.

DLBCL patients with chronic HBV infection had distinct mutation targets and tumorigenic pathways identified by genomic and transcriptomic analyses (1), studies reported that it was characterized by younger age and more advanced stage of the disease (1, 13). In our study, we did not observed any age or disease stage difference, but HBV infectious patients with DLBCL were accompanied by significantly more bone marrow involvement, indicating a more invasive course and a poor prognosis. The clinical outcomes of these patients remained controversial. Most recent studies suggested that patients with chronic HBV infection had poorer outcomes (1, 13, 14),only a few early studies showed similar ORR and OS (15, 16). In our study, CAR-T therapy was effective for patients with r/r DLBCL who failed R-CHOP and other salvage lines of immunochemotherapy, the ORR and CR rates were 74.5% and 39.2%, respectively. 6 patients with HBV infection (Group A) achieved an ORR and CR rate of 100% and 66.7% respectively, indicating that HBV status did not impact the efficacy of CAR-T therapy, the same as previously small-scale reports (7, 8). Based on the above results, we propose that CAR-T therapy may overcome the unfavorable prognostic factor of HBsAg positivity, but this view remains to be confirmed by further studies.

HBV reactivation can occur in patients with chronic or resolved HBV infection when receiving intensive immuno-chemotherapies. Increased awareness and antiviral prophylaxis have significantly reduced the rate of HBV reactivation. Previous studies revealed that about 5.3-20% of HBsAg positive patients suffered HBV reactivation with antiviral prophylaxis after CAR-T therapy (7–9). Nucleoside analogues (NAs) entecavir or tenofovir was now recommended standard agent for the prevention and treatment of HBV reactivation in patients under immunosuppressive therapy including CAR-T cell therapy (17, 18). In our study, all HBsAg positive patients received first-line entecavir based agent, one suffered HBV reactivation, revealing an incidence of 16.7%. Tenofovir has been reported to be more effective than entecavir in patients with positive HBeAg (19) and can rescue drug resistance during entecavir therapy (20). The optimal preventive strategy for DLBCL patients with positive HBsAg underwent CAR-T therapy is still under investigation. In resolved HBV infection patients, according to our observation, only one experienced controllable HBV reactivation during chemotherapy and no one experienced HBV reactivation after CAR-T therapy. Therefore, for this cohort, close and regular monitoring of HBV-DNA to capture reactivation events and guide antiviral treatment could be practical and cost effective. Together, HBV infection may not be a contraindication for CAR-T therapy under proper prophylactic nucleoside therapy and regular surveillance.

We also observed some non-HBV infection patients (Group B2) newly detected HBcAb positivity with undetectable HBV-DNA, indicating occult HBV infection during treatment. This reminded us that HBV status should be closely monitored throughout the course of treatment for all patients.

A patient with liver cirrhosis developed both HBV reactivation and transformation of hepatic malignancies. In chronic viral hepatitis-related cirrhosis, the 10-year predicted cumulative incidence estimates of hepatic malignancy were only approximately 4.0% (21). The exact mechanism of hepatic malignancy transformation has not yet been defined and persistent HBV infection may play a direct role by triggering specific oncogenic pathways, stimulating host immune response, and induce immune imbalance as well as driving chronic liver necro-inflammation et al (22, 23). CAR-T therapy may worsen the highly complexed immune imbalance, but in our opinion, it was not the main culprit for hepatic malignancy.

Given the retrospective nature of this study, randomized controlled groups and more cases of HBV chronic infectious patients are required in future studies. In summary, this study showed that CAR-T therapy was applicable and effective in r/r DLBCL with different HBV status. Anti-HBV treatment was highly necessary for patients with chronic HBV infection, while guided antiviral treatment with HBV-DNA monitoring could be explored in patients with resolved HBV infection.

## Data availability statement

The original contributions presented in the study are included in the article/**Supplementary Material**. Further inquiries can be directed to the corresponding author.

## Ethics statement

The studies involving human participants were reviewed and approved by the Ethics Committee of the First Affiliated Hospital of Soochow University. The patients/participants provided their written informed consent to participate in this study. Written informed consent was obtained from the individual(s) for the publication of any potentially identifiable images or data included in this article.

## Author contributions

DK and XG collected, analyzed data and wrote the manuscript. RZ analyzed data, CQ, NP and PW provided clinical care to the patient. CQ, ZJ and DW identified, consented and treated the patient as principal investigator. All authors contributed to the article and approved the submitted version.
