# Supplementary material for: Efficacy and safety of chimeric antigen receptor T cell therapy in relapsed/refractory diffuse large B-cell lymphoma with different HBV status: a retrospective study from a single center
Source: Front Immunol. 2023 May 24;14:1200748. doi: 10.3389/fimmu.2023.1200748 (PMC10246493; doi:10.3389/fimmu.2023.1200748)
Supplement: Supplementary file 1 [file DataSheet_1.docx]

**S1 CAR-T Cell Preparation steps**

The peripheral blood lymphocytes were acquired through density gradient separation from patients’ peripheral blood collected by apheresis. T lymphocytes were further collected and separated from peripheral blood lymphocytes by degradable anti-CD3 magnetic microbeads (Miltenyi Biotec,

Bergisch Gladbach, Germany) and activated with 5 μg/mL CD3 and CD28 (Miltenyi Biotec) for 18–24 hours. Thereafter, the T cells were transduced with recombinant lentiviral vectors, containing the anti-CD19/22 single-chain variable fragment (scFv), the cytoplasmic portion of the 4-1BB costimulatory moiety, and the CD3z T-cell activation domain, by adding lentivirus directly to the medium. After 48 hours of lentiviral transduction, the cells were washed and expanded with the complete medium (X-vivo 15 medium; Lonza, GA) containing 5 ng/mL interleukin (IL)-7 (Peprotech, NJ,USA) and 5 ng/mL IL-15 (Peprotech, NJ) cytokines under a 37°C, 5% CO2 humidified condition and maintained at a concentration of 0.5×10^6^ cells/mL for 12–20 days until their numbers met the preset value. Finally, the second-generation CAR-T cells targeting CD19, CD20 or CD22 were harvested, washed with 0.9% saline, and concentrated in cryopreservation followed by quality tests including cell viability, transduction efficiency, purity, killing ability in vitro, cytokine release capacity, quantity of endotoxin, mycoplasma, bacteria, fungus, and so on before infusion to patients. Acceptance criteria for CAR-T cell infusion were as follows: (1) Trypan blue viability > 90%, (2) CAR transduction efficiency > 10%, (3) purity of CD3+ cell > 95%, CD19+ cell: negative, (4) killing ability in vitro > 10%, (5) cytokine release capacity > 10%, (6) Gram stain: negative, endotoxin <3 EU/mL, (7) mycoplasma (polymerase chain reaction): negative, and (8) fungus: negative. In this study, the amplified folds of total cell number ranged from 50 to 100, and the proportion of CAR expressed cells in the final cell products ranged from 30% to 70%. All these CAR-T products were provided by the UnicarTherapy Bio-medicine Technology Co.

**S2 CAR-T cells infusion for 51 r/r DLBCL patients**

| Patient NO. | Conditioning regimen | Target | CAR-T cell infusion dosage(10E7/kg) |
| --- | --- | --- | --- |
| A-1 | FC | CD19+CD22 | CD19(1.5×10E7/kg);CD22(2×10E7/kg) |
| A-2 | FC | CD19+CD22 | CD19(1.5×10E7/kg);CD22(2×10E7/kg) |
| A-3 | FC | CD19+CD22 | CD19(1.5×10E7/kg);CD22(2×10E7/kg) |
| A-4 | DAC+FC | Tandem CD19/CD22 | 1×10E7/kg |
| A-5 | DAC+FC | Tandem CD19/CD22 | 1×10E7/kg |
| A-6 | DAC+FC | Tandem CD19/CD22 | 1×10E7/kg |
| B-7 | FC | CD19 | 1×10E7/kg |
| B-8 | FC | CD19 | 1×10E7/kg |
| B-9 | DAC+FC | Tandem CD19/CD22 | 1×10E7/kg |
| B-10 | FC | CD19 | 1×10E7/kg |
| B-11 | FC | CD19 | 1×10E7/kg |
| B-12 | DAC+FC | Tandem CD19/CD22 | 1×10E7/kg |
| B-13 | DAC+FC | Tandem CD19/CD22 | 1×10E7/kg |
| B-14 | DAC+FC | Tandem CD19/CD22 | 1×10E7/kg |
| B-15 | FC | CD19+CD20 | CD19(1×10E7/kg);CD20(1.7×10E7/kg) |
| B-16 | FC | CD19+CD22 | CD19(2×10E7/kg);CD22(1.5×10E7/kg) |
| B-17 | FC | CD19+CD20 | CD19(2×10E7/kg);CD20(2×10E7/kg) |
| B-18 | DAC+FC | Tandem CD19/CD22 | 1×10E7/kg |
| B-19 | DAC+FC | Tandem CD19/CD22 | 1×10E7/kg |
| B-20 | DAC+FC | Tandem CD19/CD22 | 1×10E7/kg |
| B-21 | DAC+FC | Tandem CD19/CD22 | 1×10E7/kg |
| B-22 | DAC+FC | Tandem CD19/CD22 | 1×10E7/kg |
| B-23 | FC | CD19+CD20 | CD19(2.2×10E7/kg);CD20(2.8×10E7/kg) |
| B-24 | FC | CD19 | 0.2×10E7/kg |
| B-25 | DAC+FC | Tandem CD19/CD22 | 1×10E7/kg |
| B-26 | - | Tandem CD19/CD22 | 1×10E7/kg |
| B-27 | DAC+FC | Tandem CD19/CD22 | 1×10E7/kg |
| B-28 | DAC+FC | Tandem CD19/CD22 | 1×10E7/kg |
| B-29 | FC | CD19+CD22 | CD19(1.5×10E7/kg);CD22(1×10E7/kg) |
| B-30 | DAC+FC | Tandem CD19/CD22 | 1×10E7/kg |
| B-31 | DAC+FC | Tandem CD19/CD22 | 1×10E7/kg |
| C-32 | DAC+FC | CD19 | 1×10E7/kg |
| C-33 | FC | CD19+CD20 | CD19(1×10E7/kg)+CD20(0.4×10E7/kg) |
| C-34 | FC | Tandem CD19/CD22 | 1×10E7/kg |
| C-35 | FC | Tandem CD19/CD22 | 1×10E7/kg |
| C-36 | DAC+FC | Tandem CD19/CD22 | 1×10E7/kg |
| C-37 | FC | CD19+CD22 | CD19(2×10E7/kg);CD22(1.5×10E7/kg) |
| C-38 | FC | CD19+CD22 | CD19(1.5×10E7/kg);CD22(1×10E7/kg) |
| C-39 | FC | CD19+CD20 | CD19(1.5×10E7/kg);CD20(3.5×10E7/kg) |
| C-40 | FC | CD19+CD20 | CD19(1.4×10E7/kg);CD20(1.2×10E7/kg) |
| C-41 | FC | CD19+CD22 | CD19(2×10E7/kg);CD22(1.5×10E7/kg) |
| C-42 | DAC+FC | Tandem CD19/CD22 | 1×10E7/kg |
| C-43 | DAC+FC | Tandem CD19/CD22 | 1×10E7/kg |
| C-44 | DAC+FC | Tandem CD19/CD22 | 1×10E7/kg |
| C-45 | DAC+FC | Tandem CD19/CD22 | 1×10E7/kg |
| C-46 | FC | CD19 | 0.13×10E7/kg |
| C-47 | FC | CD19 | 0.2×10E7/kg |
| C-48 | DAC+FC | Tandem CD19/CD22 | 1×10E7/kg |
| C-49 | FC | CD19+CD20 | CD20(1×10E7/kg);CD19(1×10E7/kg) |
| C-50 | FC | CD19+CD22 | CD19(1×10E7/kg);CD22(1.5×10E7/kg) |
| C-51 | DAC+FC | Tandem CD19/CD22 | 1×10E7/kg |

DAC:  decitabine; FC: fludarabine + cyclophosphamide


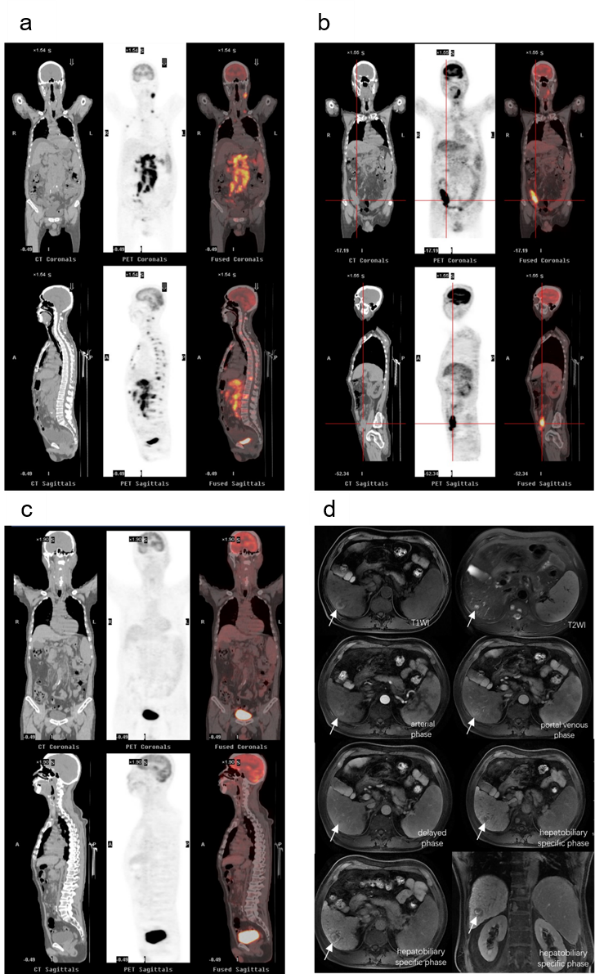


Fig1 Major radiographic assessments of the DLBCL patient with cirrhosis during treatment. (A) PET-CT scan at diagnosis showed high SUV of duodenum, multiple enlarged lymph nodes and bones. (B) PET-CT after R-CHOP showed less involved lymph nodes and bones, but newly discovered invasion of terminal ileum. (C) PET-CT after CAR-T cell therapy showed complete metabolic response of DLBCL. (D) Enhanced MRI by Gd-EOB-DTPA for the diagnosis of liver malignant tumor(white arrows).
